# Mapping patient journeys: Exploring patient and informal carer experiences of injectable anticipatory medication care in the community to identify opportunities for practice improvements

**DOI:** 10.1177/02692163261437596

**Published:** 2026-05-05

**Authors:** Rosanna Fennessy, Artemis Paterson, James Ward, P. John Clarkson, Ben Bowers

**Affiliations:** 1 Primary Care Unit, Department of Public Health and Primary Care, University of Cambridge, UK; 2 Department of Engineering, University of Cambridge, UK

**Keywords:** patient navigation, anticipatory prescribing, palliative medicine kit, terminal care, palliative care, patients, informal caregivers, user-centred design

## Abstract

**Background:**

Injectable anticipatory medications are routinely prescribed ahead of need in many countries to help manage distressing end-of-life symptoms. However, little is known about the lived experience of patients and informal caregivers as they navigate their prescription, supply and use.

**Aim:**

To explore and map patient journeys in navigating anticipatory medication care, and to identify healthcare interactions with the greatest potential for enhancing patient and informal caregiver experiences of care.

**Design:**

Qualitative secondary analysis of longitudinal interview data using framework analysis and patient journey mapping techniques.

**Setting/participants:**

Adults (18+) prescribed anticipatory medications (*n* = 6), informal caregivers (*n* = 9) and health care professionals involved in their care (*n* = 5).

**Results:**

Visually mapping journeys highlighted that patients and informal caregivers’ experiences of anticipatory medication processes varied greatly and were influenced by the context of care. All participants appreciated access to injectable medications for future symptom control. However, journeys repeatedly highlighted suboptimal information exchange between patients, informal caregivers and healthcare professionals, regarding their purpose and threshold for use. Navigating unfamiliar and complex end-of-life medication support systems was more challenging when patients lived alone or experienced communication difficulties.

**Conclusions:**

Patient and informal caregiver experiences of timely symptom control could be improved by healthcare professionals having open and ongoing conversations about the role of anticipatory medications. Simplified and well-signposted routes for accessing healthcare professional advice and medication input are needed. Using journey mapping offers a novel way to visually illustrate different patient and informal caregivers lived experience and can be adapted for researching experiences of various care pathways.

**What is already known about the topic?**

- Anticipatory prescribing is considered best practice in aiding the timely control of distressing end-of-life symptoms in the community.
- The process of prescribing, supplying and administering injectable anticipatory medications in the community is complex, with many decision-making points and practical activities required to join up care.

**What this paper adds?**

- Our patient journeys highlight how individual situations and advocacy skills greatly influence patient and families’ experiences of anticipatory medication care.
- Patients and informal caregivers find the systems for using anticipatory medications complex and clear professional signposting is needed.

**Implications for practice, theory or policy**

- Open and ongoing conversations about the purpose and use of anticipatory medications need to be tailored to individual patient and informal caregivers’ contexts.
- Journey mapping techniques are an innovative method for understanding patients’ and their families’ experiences and can be used to improve cross-organisational systems for delivering end-of-life care.

## Background

Injectable anticipatory medications are routinely prescribed ahead of need to help manage distressing end-of-life symptoms in the community.^1,2^ This is considered best practice in many countries, and as increasing numbers of people choose to die at home, their wider availability and use is promoted in policy and practice.^3–6^ Anticipatory medications are prescribed to alleviate symptoms of breathlessness, pain, nausea and vomiting, agitation and respiratory secretions,^7,8^ and to prevent unwanted emergency hospital admissions.^3,6,9^ They are typically prescribed by general practitioners (family physicians), collected from the pharmacy by family caregivers and stored at the patient’s home. The timing of prescription varies greatly, ranging from days to months ahead of likely need.^10–12^ This extended time frame is largely due to challenges of predicting end-of-life trajectories, particularly for people with non-cancer conditions such as multimorbidity and frailty.^13–15^ Decisions to subsequently administer medications are typically made by visiting community based nurses or paramedics.^16–18^ Anticipatory medications are used in approximately 60% of cases where they are prescribed, and are first started a median of 3 days before death.^16^

Research has highlighted that patients and informal caregivers find having these injectable medications in the home reassuring, but they are also a cause of concern as they can be viewed as a harbinger of death.^6,17,19,20^ Informal caregivers also express concerns about the risks of medication causing over-sedation and hastening death.^6,19^ The steps in the intended (typical) injectable anticipatory medication pathway include prescribing, dispensing, storage, medication administration (use) and disposal after death. These processes often involve multiple interactions with different healthcare providers and the sharing of information across organisations. Healthcare professionals can find it challenging to navigate anticipatory medication processes to ensure patients receive timely symptom control.^2,11,17^ Prescribing errors, incomplete permission to administer charts, pharmacy supply difficulties and delays in timely administration are recurring issues.^16,18,21,22^However, little is known about how patients and informal caregivers, in varying contexts, experience negotiating the processes of prescribing, supply, and administration of anticipatory medications.^2^

Patient and informal caregiver experiences of navigating health care systems can be visualised using journey mapping techniques. These are rooted in systems approaches from engineering and inclusive design^23–26^ and are used to help improve safety and systems by mapping patient-centred journeys and interactions with healthcare services. They help shift the focus onto service users’ diverse needs and capabilities when (re)designing healthcare systems.^26–28^ The approach helps to challenge care providers assumptions about how care is navigated and experienced. Tools include mapping ‘touch points’ (where patients and caregivers interact with healthcare professionals) and ‘pain points’ (where indivdiduals experience challenges navigating healthcare systems)^24,26,29–31^ (Table 1). These journey mapping techniques remain underused in healthcare research and can aid learning from lived experiences as people navigate different care pathways.

**Table 1.** Journey mapping concepts adopted in the present study.

Journey mapping concepts	Description and references
Touch point	Touch points represent contact between a patient/caregiver and any aspect of a service.^24,26,31^ In the current study this included any interactions with healthcare professionals related to anticipatory medication or associated end-of-life care.
Pain point	Pain points encompass aspects of a service journey which service users find challenging.^24,30,31^ In the current study this included interactions with healthcare professionals or associated systems such as sourcing medication supplies.
Adaptation	Adaptation describes how systems ‘evolve’ or are changed by actors within them.^32,33^ In the present study this included where patients and informal caregivers adapted anticipatory medication care processes.

Our study aimed to explore and map patient journeys in navigating anticipatory medication care, and to identify the healthcare interactions with the greatest potential for enhancing patient and informal caregiver experiences of care.

## Methods

### Design

We conducted a secondary qualitative analysis of longitudinal, multi-perspective interview data originally collected to explore views and experiences of anticipatory medication care.^19,34^ This dataset included rich insights into patient experiences over time from multiple perspectives but was underutilised in the original cross-case thematic analysis.

The secondary analysis was guided by a constructivist approach, acknowledging that participants’ views and experiences are shaped through interactions and shared social worlds.^35^ We used an abductive framework analysis and journey mapping techniques to visualise our findings related to patient and informal caregiver experiences of anticipatory medication care.

### Public involvement

Our Public and Clinician Advisory Group which includes family carers and community nurses with experience of anticipatory medication care were involved throughout. They helped in interpreting the relevance of the findings and selecting key aspects of care to focus on in the final visualisations of the patient journeys. PPI is reported using the GRIPP2 checklist.^36^

### Ethical approval

The South Cambridgeshire Research Ethics Committee (reference: 19/EE/0361) (01/2020) granted approval for the original study, including the use of anonymised data for this secondary analysis.

### Setting

The original research took place in two counties in England including urban and rural areas with variations in affluence.

### Population

Eligible participants were patients aged 18+ years, prescribed injectable anticipatory medications prior to recruitment, and living at home or in a residential care home. Eligible informal caregivers were family members or friends. Healthcare professionals with a key role in the patient’s anticipatory medication care were nominated by patient and/or informal caregiver participants.

### Sampling

Purposive sampling was used in the original study to recruit patients from different living circumstances, with a range of terminal health conditions and across varying ages. The sample size was informed by the principles of information power.^37^

### Recruitment

In the original study clinical teams involved in anticipatory medication care judged which patients and/or informal caregivers were suitable for the researchers to contact. Participants were contacted by the original researcher (BB). Written informed consent was obtained before first interviews, and ongoing consent confirmed prior to subsequent interviews. Participants also consented during the original study to allow anonymised data to be used for further research. One general practitioner did not consent to this, and their data has been excluded from this secondary analysis.

### Data collection

The dataset comprised longitudinal data from multiple interviews collected between May and December 2020 by a clinical academic community nurse (BB). Eleven Patient cases were recruited. A patient case comprised a patient and/or their informal caregivers and health care professionals involved in their anticipatory medication care. In some cases, both the patient and informal caregiver chose to take part, in others it was the patient or informal caregiver only. Healthcare professionals were nominated by patients or informal caregivers. However, not all health professionals agreed to take part. Interviews were undertaken by phone. Up to three interviews per case were offered, the first on study recruitment, followed by a second 2–3 months later (sooner if anticipatory medications were used). In cases where the patient died, a third follow-up interview was offered to informal caregivers 3 months after bereavement. Interview guides explored participants’ perspectives on the prescribing and use of anticipatory medications, in addition to preferences for future care (Supplemental File 1). One-off interviews were undertaken with healthcare professionals with a topic guide related to their views and experiences of anticipatory medication care in the patient’s case (Supplemental File 1). All data was anonymised before use in the current study.

### Data analysis

The qualitative data analysis and journey mapping visualisations comprised six sequential stages:

1. Anonymised interview transcripts from the original study were analysed using a framework analysis approach.^38,39^ This was chosen because it is a method suited to abductive analysis. Analysis was undertaken by RF, a research psychologist, AP, a medical student and BB, who conducted the original interviews. We inductively created codes related to patient context, and experiences of anticipatory medications and associated end-of-life care, but also deductively coded the data looking for the journey mapping concepts – touch points, pain points and adaptations. Using NVivo 14, RF and AP initially coded the same three cases independently and iteratively refined the coding framework through reflexive group discussions with the wider study team. Remaining transcripts were then coded, and the framework adapted as new codes were identified.
2. Interviews from the 11 patient cases were systematically compared across codes and we selected three cases (Sue, Ted and Liam) to produce visual journeys. These were chosen to highlight a range of circumstances and experiences of anticipatory medication care evident across the 11 patient cases. These included shorter or longer end-of-life trajectories, experiences of those living alone or with family and differences in advocacy capabilities. While journey mapping techniques often use fictional ‘personas’ (amalgams of characteristics designed to represent particular groups), we chose real participants’ journeys as we wanted to illustrate lived experience and use verbatim quotations.
3. Together, RF, AP and BB visually mapped the chosen three patients’ journeys highlighting touch points, pain points and adaptations, alongside the key processes in the anticipatory medication pathway (prescribe, dispense, store, use, dispose), to illustrate similarities or differences in experience (Supplemental File 2). By visualising participants’ experiences we identified three recurring factors which affected anticipatory medication care journeys. In addition to the visual journeys, we returned to our framework codes and the primary data to confirm these recurring factors were also evident across the dataset. We created summaries of the remaining eight cases through this process (Supplemental File 3).
4. Pseudonyms were used throughout, and health and social information further selectively modified to protect anonymity.
5. Grounded in verbatim quotations, we created a condensed narrative for Sue, Ted and Liam to draw out the recurring factors of interest, key touch points, pain points and adaptations.
6. Our three visualised journeys were professionally illustrated.

## Results

### Participant details

Across the eleven patient cases, six patients, nine informal caregivers and five healthcare professionals took part over an 8-month period (27 interviews in total; Table 2). Injectable anticipatory medications were prescribed between 294 and 5 days before patient death or the final interview in the case. Medications were used (administered) in seven of the eleven cases (64%).

**Table 2.** Participant details.

Patient case^c^	Age range	Gender	Participants from each case who took part with number of interviews (n)	Terminal/life limiting conditions^a^	Living arrangement	No. of days anticipatory medications prescribed before death or final interview	Medications obtained via	Medications used
Sue^b^	65–74	F	Patient: Sue (2)	Cancer	At home with family	123 days before final interview	Delivered by community nurses	Y
Ted^b^	85–94	M	Friend: Sarah (2) General practitioner: Sam (1)	Heart failure, frailty	At home alone	5 days before death	Collected by relative	N
Dave	75–84	M	Partner: Katie (2) Daughter: Zoe (2) General practitioner: Victor (1)	Heart failure, kidney failure, frailty	At home alone	184 days before final interview	Collected by relative	N
Helen	85–94	F	Daughter: Alice (1)	Heart failure, cancer	In care home	11 days before death	Delivered to care home	Y
Hugo	85–94	M	Daughter: Emily (2) Community nurse: Charlie (1)	Heart failure, frailty, cancer	In care home	113 days before final interview	Collected by relative	N
Louise	65–74	F	Patient: Louise (2)	Cancer	At home with family	191 days before final interview	Delivered by pharmacy	Y
Liam^b^	65–74	M	Patient: Liam and Partner: Amelia together (1) Partner: Amelia – after patient’s death (1)	Cancer, heart failure, frailty,	At home with family	97 days before death	Discharged from hospital with them	Y
Ruth	75–84	F	Grandson: Mark (2)	Cancer	At home with family	79 days before final interview	Delivered after family collected paperwork from GP	Y
Joe	65–74	M	Patient: Joe and Partner: Kim together (1) Palliative care nurse: Gail (1)	Cancer	At home with family	79 days before death	Collected by patient	Y
Abby	75–84	F	Patient: Abby (2)	Respiratory disease	At home with family	294 days before final interview	Collected by relative	N
Dylan	75–84	M	Patient: Dylan and Partner: Freya together (1) Partner: Freya- after patient’s death (1) Palliative care nurse: Lana (1)	Cancer	At home with family	29 days before death	Delivered to home	Y

a As reported by participants

b Cases selected to create patient journeys.

c A patient case consisted of a patient, nominated informal caregivers and nominated health professionals.

The three recurring factors which impacted patient experiences of navigating anticipatory medication care across the 11 patient cases were:

1. *Perceptions of anticipatory medications*
  Patients and informal caregivers noted the usefulness of anticipatory medications to relieve potential pain and suffering in the future. However, when medications were used, they were not always perceived as being helpful. Some patients and informal caregivers had concerns about medications causing over-sedation and potentially hastening death, others found that hospitalisation was more helpful in managing symptoms.
2. *Quality of information exchange*
  Information sharing between healthcare professionals and patients and informal caregivers shaped patient journeys, both positively and negatively. This included communication about prescribing anticipatory medications and when they might be used, along with ongoing contact and dialogue as patients’ journeys progressed.
3. *Ability to navigate healthcare systems*
  Navigating complex and unfamiliar processes related to anticipatory prescribing and end-of-life care was often problematic. Patients who could advocate for themselves or had live-in caregivers were more successful in accessing timely care, compared to those who lived alone or had less capacity to communicate.These factors are illustrated in the visual journeys of our three patients Sue, Ted and Liam -.

**Figure 1.**
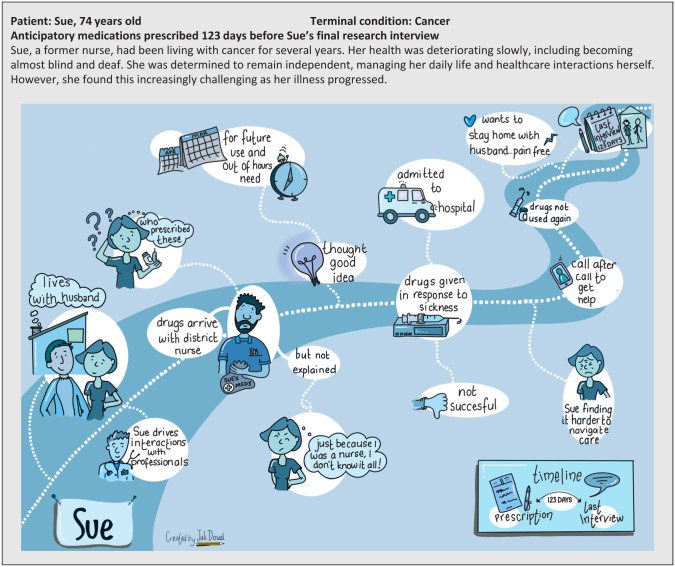
Sue’s journey. (Illustration created by Juli Dosad)

## Sue’s Journey

When Sue’s oncologist advised that care was now palliative, anticipatory medications were prescribed, although she was not sure by whom – they “just appeared” [Sue] with the community nurses. Sue read the leaflets in the packets but thought that the lack of explanation from healthcare professionals was unhelpful and might be due to assumptions about her medical background:

I don’t want people to think that I know things because I don’t. . . I do think sometimes they presume that they don’t have to say things because you’re a nurse and you should know that but it don’t work like that! [Sue]

Nevertheless, Sue was happy to have anticipatory medications in the house for potential future use:

I know what they’re all for but I think, you know, you’ve just got to have them quick, if you need them, especially at weekends and stuff like that. [Sue]

Sue advocated for herself with healthcare professionals, telling the community nurses that she would contact them if necessary. When she experienced an episode of continuing sickness, the anticipatory medications were commenced using a syringe driver. However, after a community nurse and two out-of-hours doctors attended, she was admitted to hospital. Sue subsequently felt that the anticipatory medications were not helpful:

I’d probably say, well, it just didn’t go right for me. Or perhaps I was just too far gone for anything to, I needed fluid. . . It was put up in the ambulance, I was so dehydrated. . . Having the drugs, without having fluid first, I think it was just a bit of a mess up. [Sue]

Sue’s health had deteriorated considerably by the second interview. While she wanted to remain independent and advocate for herself, she found navigating health care services increasingly challenging. The considerable work involved is illustrated by the difficulties she experienced when trying to source medication for constipation:

The bad days, just too much happening and too much messing about with phone calls, trying to get somewhere. Like Saturday morning, because I was a bit desperate with this bowel thing, I was just like, it was one thing after another, 111 [urgent health advice phoneline], through to the chemists, chemists computers are down. And then when you’re doing all this, you’re finding that, oh my goodness, I haven’t eaten lunch and I’ve got to take pills and it’s getting late. [Sue]

**Figure 2.**
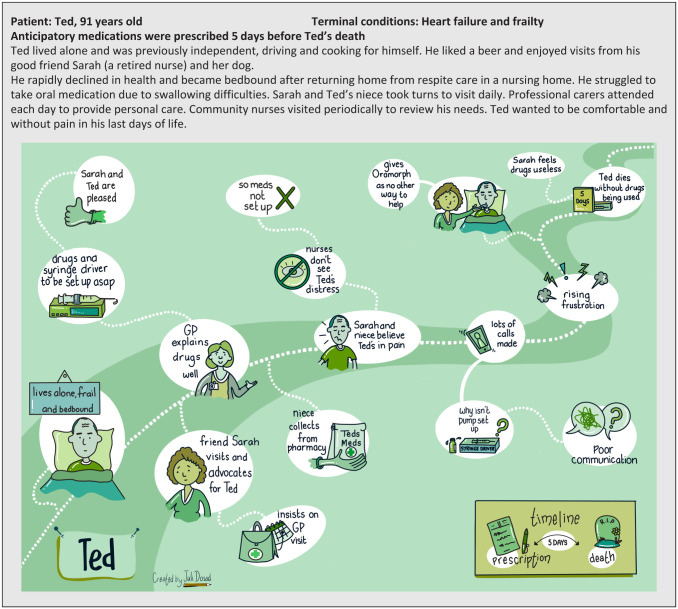
Ted’s journey. (Illustration created by Juli Dosad)

## Ted’s Journey

Sarah’s nursing background led her to insist on a general practitioner home visit for Ted to discuss his deterioration and the likelihood that he was now dying. Sarah and Ted’s niece were present at this visit and advocated on Ted’s behalf:

I felt he’s just been left and if I hadn’t had that knowledge and knew what I wanted for him or what was available for him, I don’t think he would have had it. [Sarah]

The general practitioner explained what anticipatory medications were for and prescribed them to start via a syringe pump. They believed this would be used immediately to help with Ted’s pain:

And she [the general practitioner] said, well we’re going to give you some things to make you comfortable but it’s not going to make it any quicker but it’ll make that time, hopefully, more comfortable for you. . . She implied that we would be starting it there and then because Ted wants to die, he’s uncomfortable, he was being sick, and he was happy to start that pump there and then [Sarah]

Sarah thought the syringe pump would be particularly useful for managing Ted’s nausea and chest secretions:

Well to me the pump’s just going to make him more comfortable, and he keeps trying to be sick, we try and give him things and he’s had a lot of secretion that he keeps coughing up, you know what I mean, and it will hopefully just let him rest and have nice dreams and not be uncomfortable. [Sarah]

The general practitioner identified a pharmacy with injectable medication stock and Ted’s niece collected them. However, they weren’t given any further details of the drugs or next steps. The syringe pump was not set up and no medication was given by the nurses:

I think there should have been some sort of conversation with us about them, we weren’t given a leaflet about it, or how it would all be set up, we weren’t informed of anything like that. So, because we were led to believe by the GP that the palliative nurses will come up and set this pump up and it’ll be nice and relaxed and that, that’s what we were thinking would happen which it never did. [Sarah]

Sarah and Ted’s niece found getting information from Ted’s healthcare team challenging and were distressed that they could not persuade the community nurses to start the anticipatory medications. Their daily visits did not coincide with the nurses, limiting opportunities to communicate:

We’ve phoned the palliative nurses but what you do, you go through a call centre or whatever. We’ve said look, he’s at home on his own, these palliative nurses are coming in, we don’t know what they’re doing. . . There’s nothing written at home to say what the plan is, so we feel we’re not in the loop. . . Why has the pump not been done and it is just very frustrating, you know, to see him like that and stuff’s there to make him more comfortable and nothing’s happening. [Sarah]

The nurses told Sarah by phone that Ted did not need the anticipatory medications. However, Sarah observed Ted in distress on her morning visits and gave him liquid morphine to make him more comfortable for when the carers arrived to wash him. She left notes for the nurses to let them know this and rationalised that maybe they did not witness his distress:

Maybe the district [community] nurses or palliative care nurses would come in, and he’d seem fine to them, because they only saw him for like a five-minute slot. But they didn’t see how he was all distressed and hallucinating and things like that, which was, it was worse for me because I was the one to find him. [Sarah]

An interview with Ted’s general practitioner highlighted differences in healthcare professionals’ perceptions over whether the injectable medications should have been started:

I prescribed it so it could be delivered by syringe driver as well if needed. And I sort of, I thought that was going to be the way to go. . . but we refer to the district [community] nurses. . . they did go and visit him twice a day, but they didn’t think a driver was necessary. So that was a slight discrepancy in what I thought would happen and versus what did happen. [Sam, General practitioner]

Ted died at home 5 days after the medications were prescribed, without any of the drugs being given. Sarah considered that the anticipatory medications were “useless because nobody would give him anything” [Sarah].

**Figure 3.**
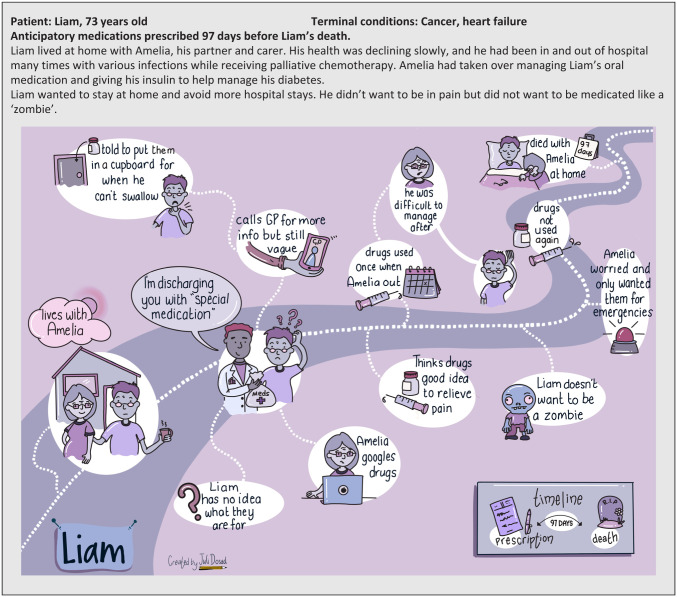
Liam’s journey. (Illustration created by Juli Dosad)

## Liam’s Journey

At the end of a period in hospital, Liam’s discharge was delayed as some “special medications” [Liam] had been prescribed but not dispensed. Liam asked questions about what turned out to be the anticipatory medications, but the hospital pharmacist did not explain them:

I said, “Well, what is this?”, and they said, “We don’t know, it’s some special medication that the doctors have to send, you have to go home with”, which apparently, learning, on reflection, is this bag of goodies [anticipatory medication]. So that was the first thing I knew about it. No one had mentioned it to me, I’d never heard of it. [Liam]

Once home, Amelia looked up the names of the medications on the internet. She also phoned their general practitioner, although she would have preferred more detail in his response:

I said to Dr Potter, “Well, I’ve got a bag here and it says Just in Case”, and he said, “Yes, put them in the cupboard for later in case Liam can’t swallow”. . ., so he sort of like skirted over but told me what they’re for. [Amelia]

Amelia advocated strongly on Liam’s behalf including his desire not to go back into hospital. They also believed the chemotherapy tablets were causing much of Liam’s pain, and managing this became a source of concern:

I’ve got. . . cancer. Which no ones used the word terminal to me, but I know it’s terminal, okay. One or two doctors have said, “Yeah, we can’t, we can’t cure it, Liam, but we just, we can control it”. . . If controlling it means suffering pain all the time, then I’m not interested in that. But then, I don’t want to go to the other side so that I’m sort of no pain but just lying like a zombie in bed, I can’t see any point in having that sort of life. So we’ve got to find a balance between the two. [Liam]

A few weeks before his death and whilst Amelia was out, a community nurse gave Liam a dose of the anticipatory medications to “calm him” [Amelia]. Amelia was upset by its impact as she was unable to manage Liam physically afterwards and was concerned about the potential for the drugs to be misused. She subsequently insisted they only be used for emergencies. Liam died at home with Amelia by his side. The anticipatory medications were not used again:

It was reassuring that if he did suffer, then I knew that all I had to do was to call a district [community] nurse and she would come out and take that suffering away from him, that was always there in my mind. . .. When I had the episode. . . I thought how easy it is for someone just to come and, in my terms, bump somebody off. So, yeah, no, they stayed in the cupboard, for purely an emergency only. [Amelia]

### Identifying interactions with the greatest potential for improvement in practice

We identified three opportunities where interactions between patients, informal caregivers and healthcare professionals offered potential for enhancing patient and caregiver experiences of anticipatory medication care.

### Explanations of anticipatory medications

Anticipatory medication prescriptions need open and tailored explanations by healthcare professionals. This includes exploring patients’ and informal caregivers’ interpretations of the purpose of medications. The three patient journeys highlight that anticipatory medications were often not adequately explained. For example, Amelia had to source information from the internet. Those with medical backgrounds had some understanding, but as noted by Sue, she did not want people to make assumptions about her knowledge. While participants commented on the usefulness of these medications for the relief of future pain, further information and discussion were often needed, as differing perceptions about the medications and their effects were commonplace.

### Optimising system transparency

Although the decision to prescribe anticipatory medications was driven by healthcare professionals, greater transparency and ongoing communication could have helped patients and informal caregivers to better navigate support systems related to their use. This was particularly notable for Ted and Sarah who didn’t understand why the medications and syringe pump were not used. The challenges of communicating with multiple healthcare teams also impacted experiences of using anticipatory medications. Sarah thought the medications were ‘useless’ due to the difference between the perceptions of the general practitioner and the nurses regarding the threshold for their use. For Amelia, anxiety regarding their potential to hasten death meant she no longer wanted them to be used. Continued dialogue and clear signposting regarding how to access help with symptom control could have helped alleviate patient and informal caregiver distress.

### Tailoring input to individual patients’ and informal caregivers’ circumstances

Our three journeys illustrate how the different circumstances and resources of patients and informal caregivers influenced their ability to navigate unfamiliar healthcare systems. Sue maintained her self-advocacy, although found things more challenging over time, while Liam and Ted relied on others. However, Amelia was more successful in advocating, perhaps because she lived with Liam. Conversely, Sarah had considerable difficulty advocating for Ted’s ongoing needs. Recognising differences in the individual resources of patients and their informal caregivers over time, along with adapting care provision to meet variable situations and needs, would improve experiences of timely symptom control care.

## Discussion

Our study provides important insights into the lived experience of anticipatory medication care. Our journey visualisations highlight how patient and informal caregiver experiences varied as they navigated the prescription, supply and use of anticipatory medications. There was also considerable variation in how far in advance of anticipated and actual need prescriptions were put in place, mirroring the findings of previous studies.^9,10,16^ Our study found patients’, informal caregivers’ and healthcare professionals’ perceptions regarding the purpose of anticipatory medication, and when they would be used, was a source of concern and frustration for some. Patients’ self-advocacy skills, alongside whether they lived with their informal caregivers, also influenced their ability to access and navigate unfamiliar and complex end-of-life care support.

### What this study adds

Having anticipatory medications available in the home is perceived to be reassuring for all involved,^2,19,40,41^ and our findings generally concur with this. However, we found that perceived gaps in information sharing regarding the purpose, safety and likelihood of use of these medications, caused concern to patients and informal caregivers, and may add to the psychological distress experienced by patients and families at the end of life.^3,42^ There is a need for open and ongoing conversations about the role and purpose of anticipatory medications to explore and address any concerns.^12,19,43^ These need to be initiated by healthcare professionals and tailored to patients and informal caregivers existing knowledge and preferences for information.

Ongoing information sharing about symptom control is important as patients and informal caregivers may not recall conversations or retain information in stressful and ever-changing situations.^19,43–45^ Information sharing at the end of life is shaped by many factors including healthcare professional concerns about causing potential distress and their confidence and experience in having these conversations.^46–49^ Not all patients and families wish to engage in explicit conversations about what the future may hold.^44,50^ However, previous research suggests patients often have a preference for open and honest communication,^44,51–53^ which reflects the desire for information found in our current study. Further research exploring generational and cultural preferences for information sharing is required.

Our patient journeys reflect differences in patient resources and self-advocacy skills; and informal caregivers became increasingly important in helping navigate anticipatory medication pathways. The work of informal caregivers is essential in meeting patients’ end-of-life care needs in the community, especially for those who live alone. They also assume significant responsibilities for managing medication and providing timely symptom control.^40,43,54–57^ However, we found that informal caregivers did not feel sufficiently involved in decisions to use anticipatory medications, despite this being found to be empowering for all concerned.^54,58^ In our current study, this could have been facilitated by providing simplified methods to contact healthcare professionals for timely clinical medication advice which would have improved participants’ experiences.

Mapping patient journeys is an important method to improve patient safety and healthcare services.^23–25^ However, the focus is often on mapping processes and work as intended rather than patient experience and priorities of navigating healthcare. By adopting ideas from inclusive design techniques, such as focusing on touch points and pain points, our novel use of secondary data enabled us to visually map patient journeys, exposing several pain points for participants as they navigated the prescription and use of anticipatory medications. This highlights the need for greater flexibility and responsiveness in existing healthcare systems. Although this method of mapping experience needs further development, such as ensuring any journeys created reflect the full range of diverse characteristics of patients, it offers rich patient-centred insights which in future research may stimulate cross-organisational improvements in care.

### Strengths and limitations

Our qualitative analysis incorporated framework analysis and journey mapping techniques which allowed for rich description and visual illustration of patient and informal caregiver experiences. Our analysis was grounded in reflexivity, and we discussed our early findings with our Public and Clinician Advisory Group to incorporate their views on the implications for practice. However, as this was a secondary analysis, we were unable to ask the original participants follow up questions.

The patient journeys represented the range of characteristics and circumstances from our sample, but these do not fully reflect the diversity of individuals who navigate end-of-life care services. We are currently seeking out unheard voices to ensure under-served populations are better represented in a new qualitative study. Similarly, while our secondary analysis incorporated data collected from healthcare professionals involved in prescribing the anticipatory medications, as this was a secondary analysis we were unable to triangulate our findings related to patient and informal caregiver experiences with the experiences of the professionals involved. The original data was collected during the Covid pandemic so there may have been unique circumstances affecting patient journeys at this time. However, studies undertaken before the pandemic and our own recent fieldwork has highlighted similar difficulties with information exchange, shared decision-making and navigating end-of-life care pathways.^2,40,45^

## Conclusions

Injectable anticipatory medications are perceived to help in providing timely symptom control for patients dying in the community. Our study demonstrated that patient and informal caregiver experiences of the prescription and use of anticipatory medications vary hugely. Exploring and visualising patient journeys helps care providers and policy makers to see the challenges experienced in navigating unfamiliar end-of-life care systems. They highlight the need for open and ongoing dialogue initiated by healthcare professionals regarding anticipatory medications, to reduce patients’ and informal caregivers’ concerns and facilitate access to timely symptom control.

## Supplemental Material

sj-docx-1-pmj-10.1177_02692163261437596 – Supplemental material for Mapping patient journeys: Exploring patient and informal carer experiences of injectable anticipatory medication care in the community to identify opportunities for practice improvements Supplemental material, sj-docx-1-pmj-10.1177_02692163261437596 for Mapping patient journeys: Exploring patient and informal carer experiences of injectable anticipatory medication care in the community to identify opportunities for practice improvements by Rosanna Fennessy, Artemis Paterson, James Ward, P. John Clarkson and Ben Bowers in Palliative Medicine

sj-docx-2-pmj-10.1177_02692163261437596 – Supplemental material for Mapping patient journeys: Exploring patient and informal carer experiences of injectable anticipatory medication care in the community to identify opportunities for practice improvements Supplemental material, sj-docx-2-pmj-10.1177_02692163261437596 for Mapping patient journeys: Exploring patient and informal carer experiences of injectable anticipatory medication care in the community to identify opportunities for practice improvements by Rosanna Fennessy, Artemis Paterson, James Ward, P. John Clarkson and Ben Bowers in Palliative Medicine

sj-docx-3-pmj-10.1177_02692163261437596 – Supplemental material for Mapping patient journeys: Exploring patient and informal carer experiences of injectable anticipatory medication care in the community to identify opportunities for practice improvements Supplemental material, sj-docx-3-pmj-10.1177_02692163261437596 for Mapping patient journeys: Exploring patient and informal carer experiences of injectable anticipatory medication care in the community to identify opportunities for practice improvements by Rosanna Fennessy, Artemis Paterson, James Ward, P. John Clarkson and Ben Bowers in Palliative Medicine
